# Epigenetic aging of human hematopoietic cells is not accelerated upon transplantation into mice

**DOI:** 10.1186/s13148-018-0499-7

**Published:** 2018-05-22

**Authors:** Joana Frobel, Susann Rahmig, Julia Franzen, Claudia Waskow, Wolfgang Wagner

**Affiliations:** 1 Helmholtz-Institute for Biomedical Engineering, Stem Cell Biology and Cellular Engineering, RWTH Aachen University Medical School, Pauwelsstraße 20, 52074 Aachen, Germany; 2 Regeneration in Hematopoiesis, Institute for Immunology, Technical University Dresden, Dresden, Germany

**Keywords:** Aging, DNA methylation, Epigenetic, Hematopoiesis, Humanized mice, Transplantation

## Abstract

**Background:**

Transplantation of human hematopoietic stem cells into immunodeficient mice provides a powerful in vivo model system to gain functional insights into hematopoietic differentiation. So far, it remains unclear if epigenetic changes of normal human hematopoiesis are recapitulated upon engraftment into such “humanized mice.” Mice have a much shorter life expectancy than men, and therefore, we hypothesized that the xenogeneic environment might greatly accelerate the epigenetic clock.

**Results:**

We demonstrate that genome-wide DNA methylation patterns of normal human hematopoietic development are indeed recapitulated upon engraftment in mice—particularly those of normal early B cell progenitor cells. Furthermore, we tested three epigenetic aging signatures, and none of them indicated that the murine environment accelerated age-associated DNA methylation changes.

**Conclusions:**

Epigenetic changes of human hematopoietic development are recapitulated in the murine transplantation model, whereas epigenetic aging is not accelerated by the faster aging environment and seems to occur in the cell intrinsically.

## Introduction

Humanized mice (HuMice) are used for a wide variety of applications in biomedical research, ranging from tumor biology, over studies of human hematopoiesis, to vaccine testing [1, 2]. Within the last decades, various mouse models have been generated to improve hematopoietic reconstitution. For example, KIT-deficient NOD/SCID *Il2rg*^*−/−*^*Kit*^*W41/W41*^ (NSGW41) mice support a stable engraftment of lymphoid and myeloid cells without the need for irradiation conditioning prior to transplantation, allowing analysis of human hematopoietic cells in a steady-state condition [3, 4]. Phenotypically, humanized mice reflect multilineage differentiation that closely resembles human counterparts. However, it was yet unclear if transplanted human cells recapitulate epigenetic changes of normal hematopoietic development. Furthermore, mice have a significantly shorter life span than men, and this might result in faster epigenetic aging upon transplantation into the faster aging cellular environment [5]. In this study, we have therefore analyzed global DNA methylation (DNAm) profiles of stably engrafted humanized mice.

## Results and discussion

**Fig. 1:**
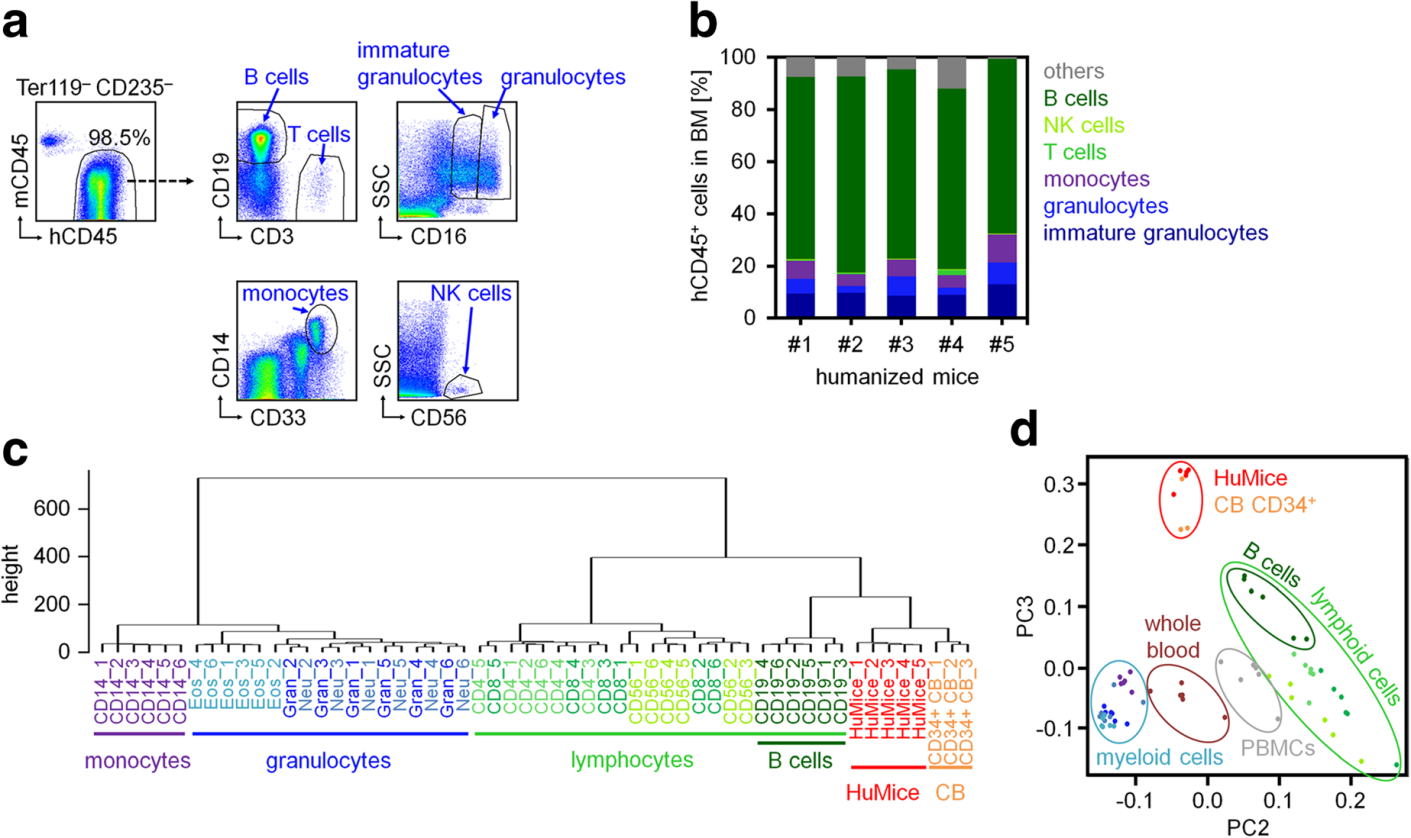
Phenotypic and epigenetic characterization of engrafted human hematopoietic cells. **a** Flow cytometric analysis of bone marrow (BM) 19 weeks after transplantation of human CD34^+^ cells into NSGW41 mice. Erythroid cells (Ter119^+^ or CD235^+^) were excluded, and human CD45^+^ (hCD45^+^) cells were analyzed for the expression of cell type-specific surface markers of B cells (CD19), T cells (CD3), monocytes (CD14), NK cells (CD56), and granulocytes (CD16). **b** Cellular composition of hCD45^+^ cells in BM of five humanized mice. Cells described as “others” include stem and progenitor cells, myeloid progenitors, and dendritic cells. **c** Unsupervised hierarchical clustering of global DNA methylation (DNAm) profiles of various hematopoietic cell types purified from peripheral blood (monocytes, granulocytes, and lymphocytes; GSE35069) or umbilical cord blood (CB; GSE40799) compared to those of hCD45 sorter purified cells from BM of humanized mice (HuMice; GSE103010). **d** Principal component analysis (PCA) of the same hematopoietic subsets described in **c**. PBMCs, peripheral blood mononuclear cells

Hematopoietic stem and progenitor cells (CD34^+^) were isolated from human umbilical cord blood (CB) and transplanted into five NSGW41 mice [6]. Nineteen weeks after transplantation, the bone marrow (BM) was harvested and flow cytometric analysis revealed that 96.4 ± 1.9% of hematopoietic cells were of human origin. Immunophenotypic analysis of these human CD45^+^ (hCD45^+^) cells reflected differentiation toward lymphoid (B cells, T cells, and NK cells) and myeloid lineages (monocytes, granulocytes, and immature granulocytes; Fig. 1a). The majority of the engrafted human cells expressed CD19 and therefore seemed to be committed toward B cell development (71 ± 3%; Fig. 1b). We analyzed genome-wide DNAm patterns of sorted hCD45+ cells with Infinium HumanMethylation450 BeadChips. In comparison to DNAm profiles of various mature human hematopoietic subsets (GSE35069) [7], unsupervised hierarchical clustering (Fig. 1c) and principal component analysis (PCA; Fig. 1d) demonstrated that epigenetic profiles of HuMice were overall still closely related to CD34^+^ CB cells (GSE40799) [8]. This was somewhat unexpected, because the engrafted cells clearly reflect immunophenotypic changes of hematopoietic differentiation.

**Fig. 2:**
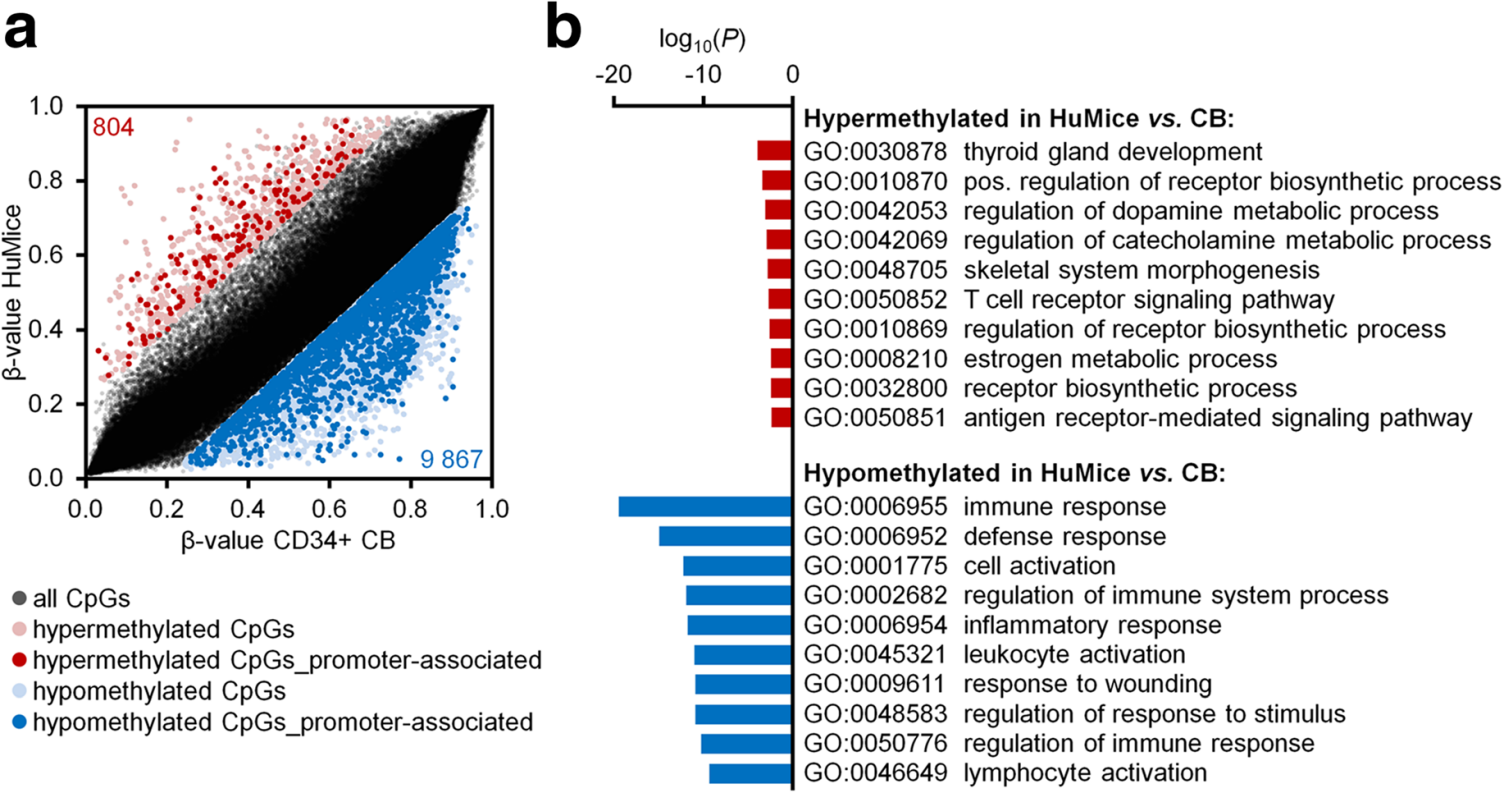
DNA methylation changes in human hematopoietic cells upon stable engraftment into mice. **a** Scatterplot of DNAm levels in humanized mice (HuMice) versus CD34^+^ cord blood (CB) samples. Significant hyper- and hypomethylation is highlighted in red and blue, respectively (delta of mean *β* values > 0.2 or < − 0.2; adjusted limma *P* value < 0.05). CpG sites that are associated with promoter regions (located in the 5′ untranslated region (5′ UTR), 200 bp (TSS200), and 1500 bp (TSS1500) upstream of transcription start site) [24] are more likely to be reflected in differential gene expression and were therefore highlighted in bold (2425 CpGs and 169 CpGs, respectively). **b** Gene ontology (GO) analysis of genes associated with differentially methylated CpG sites in promoter regions (one-sided Fisher’s exact *P* value). The most significant categories are exemplarily depicted (categories comprising more than 1000 genes were not considered and similar categories are only listed once)

To gain further insights into epigenetic changes of stably engrafted hematopoietic cells, we filtered for CpG dinucleotides with significant DNAm changes in HuMice versus CD34^+^ CB samples (adjusted *P* value < 0.05): 9867 and 804 CpGs were hypo- and hypermethylated, respectively (Fig. 2a). For functional classification, we focused particularly on genes with significantly differentially methylated CpGs in promoter regions: gene ontology (GO) analysis revealed highly significant enrichment of DNAm changes in hematopoietic categories (Fig. 2b), indicating that DNAm changes upon engraftment in HuMice are particularly associated with hematopoiesis and immune response.

**Fig. 3:**
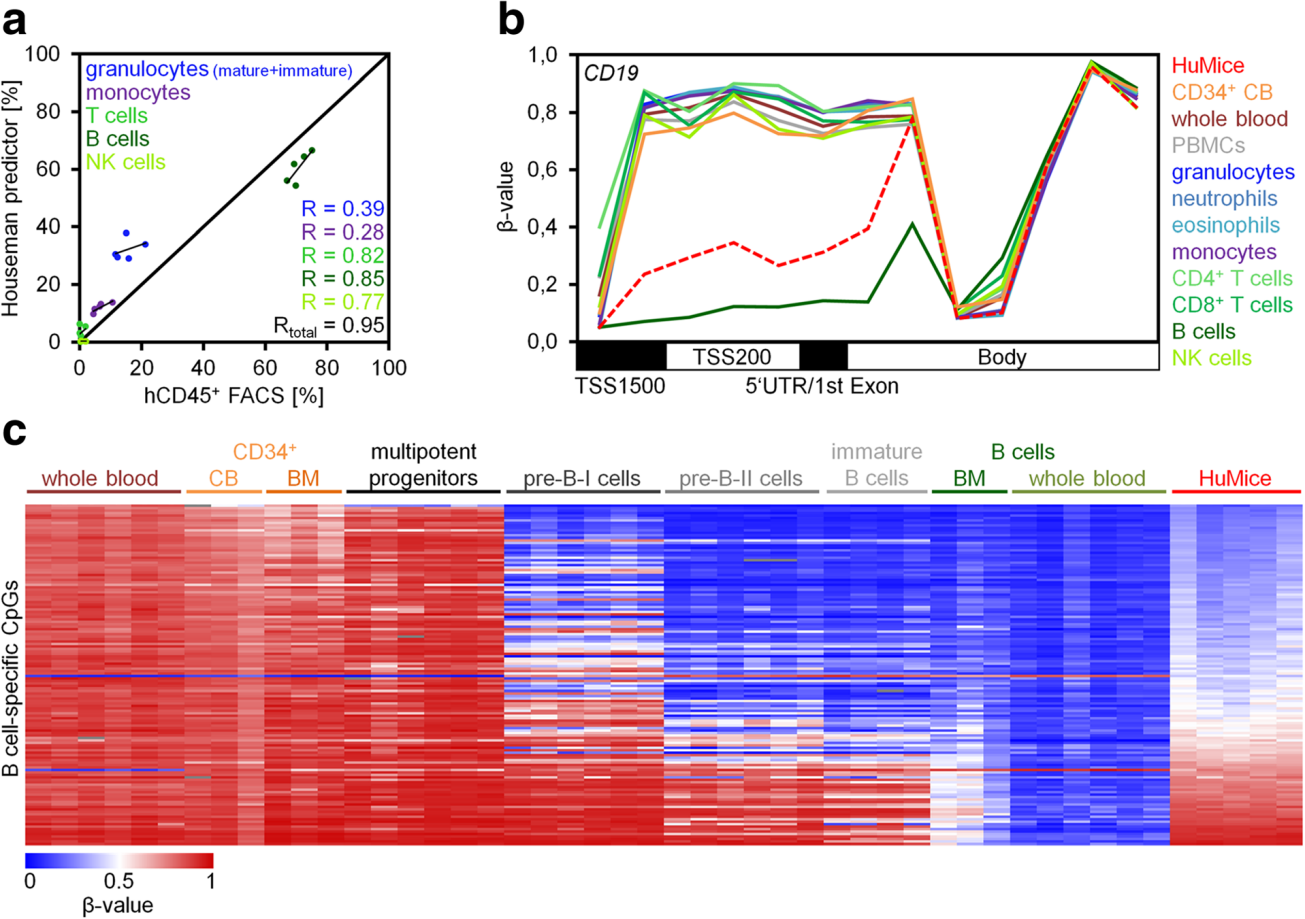
Lineage-specific DNA methylation changes. **a** Correlation of flow cytometric analysis of human CD45^+^ (hCD45^+^) bone marrow (BM) cells compared to results of a deconvolution algorithm to estimate the composition of cell types based on DNA methylation (DNAm) profiles (Houseman predictor) [9]. *R*, Pearson correlation coefficient. **b** DNAm levels (*β* values) of CpGs within the gene *CD19* for various hematopoietic subsets from peripheral blood (GSE35069), umbilical cord blood (CB; GSE40799), and from BM of humanized mice (HuMice; GSE103010). **c** Heatmap of 330 CpGs with highest differential DNAm in B cells as compared to other mature cell types (GSE35069, delta of mean *β* values > 0.7 or < − 0.7; SD < 0.1). DNAm profiles of B cell precursors were subsequently included for comparison (GSE45459; multipotent progenitors = CD34^++^CD19^−^; Pre-B-I cells = CD34^+^CD19^+^; Pre-B-II cells = CD34^−^CD19^+^sIgM^−^; immature B cells = CD34^−^CD19^+^sIgM^+^; sIgM = surface IgM). The heatmap is sorted by mean DNAm levels in HuMice

The cellular composition of hematopoietic subsets can be estimated based on DNAm patterns by deconvolution algorithms [9, 10]. When we applied the algorithm of Houseman et al. on DNAm profiles of HuMice, the estimated relative cell counts were overall in line with immunophenotypic assessment (Fig. 3a; *R*_total_ = 0.95). Furthermore, consistent with the high CD19^+^ content of engrafted cells, the promoter region of *CD19* was hypomethylated in engrafted cells with a very similar DNAm pattern as observed in sorted B cell populations from whole blood (Fig. 3b). In analogy, such characteristic patterns of B cells were also reflected in many other genes of B cell development. These results demonstrate that lineage-specific epigenetic profiles of normal human hematopoietic differentiation, particularly those of the B cell lineage, are recapitulated upon transplantation into mice.

To better understand if DNAm patterns of normal human B cells are generally acquired in HuMice, we filtered for B cell-specific CpG sites [10]. The vast majority of these CpGs were hypomethylated in B cells, and most of these were also hypomethylated in HuMice. In fact, the DNAm pattern of HuMice in these B cell-specific CpGs was perfectly in line with those of immature B cells in normal human development (GSE45459; isolated from fetal BM; Fig. 3c) [11]. These findings further substantiate the notion that the majority of engrafted cells resemble early B cell progenitor cells, which might contribute to their abovementioned close epigenetic relationship with CD34^+^ progenitor cells. On the other hand, the complex epigenetic modifications associated with lineage-specific differentiation are also initiated in the xenogeneic transplantation model.

**Fig. 4:**
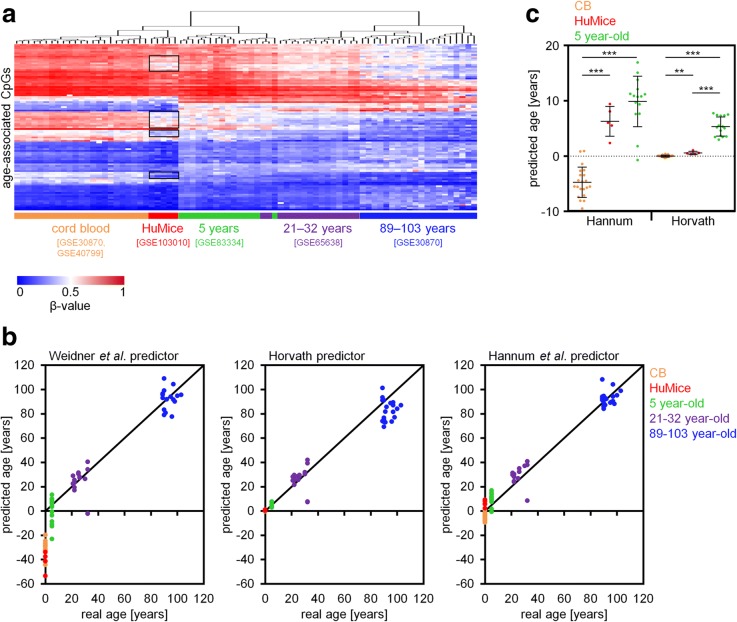
Epigenetic age predictions based on three different aging signatures. **a** The clustered heatmap represents DNAm of 99 age-associated CpG sites of the epigenetic age predictor of Weidner et al. [14]. Black rectangles highlight DNAm patterns of CpGs that may reveal moderate epigenetic age acceleration in HuMice. **b** Three epigenetic aging predictors from Weidner et al. [14], Horvath [16], and Hannum et al. [15] were used to predict donor age in 23 cord blood samples (CB; GSE40799, GSE30870), 15 whole blood samples of 5 year-old donors (GSE83334), 16 whole blood samples of 21–32 year-old donors (GSE65638), 20 peripheral blood mononuclear cell samples of 89–103 year-old donors (GSE30870), and the five samples from humanized mice (HuMice; GSE103010). The age predictor of Weidner et al. was not trained for CB and underestimates CB and HuMice samples to negative ages. **c** Epigenetic age predictors of Hannum et al. [15] and Horvath [16] were applied on DNAm profiles of CB cells, BM of HuMice, and peripheral blood of 5-year-old individuals. **P < 0.01; ***P < 0.001 (two-tailed Student’s *t* test)

It is generally anticipated that the microenvironment, the so-called stem cell niche, has a major impact on declining stem cell function in the elderly [12]. In fact, age-associated DNAm changes are acquired faster in short-lived mice than men [5, 13]. With an average murine life expectancy of about 2 years, the 19 weeks after transplantation might correspond to 15 years of human aging. To address the question if epigenetic aging is accelerated in the xenogeneic transplantation setting, we initially focused on 99 age-associated CpGs that revealed high correlation with chronological age in blood [14]. In HuMice, most of these age-associated CpGs maintained the DNAm patterns of CB, even though some of them revealed moderate changes as observed upon aging (Fig. 4a). The corresponding age predictor was not trained for CB samples, and age predictions were therefore underestimated for CB samples and HuMice (Fig. 4b). Alternatively, we used the age predictors of Hannum et al. [15] and Horvath [16], and both models consistently indicated that 19 weeks after transplantation, epigenetic aging is only moderately increased in mice (mean epigenetic age increase of 6 and 0.7 years, respectively; Fig. 4c).

Hematopoietic differentiation is governed by complex epigenetic mechanisms, which have to be triggered by the microenvironment. In this study, we demonstrate that the xenogeneic milieu of a murine transplantation model evokes very similar DNAm changes as observed in human hematopoiesis. On the other hand, the epigenetic makeup seems to be stalled on progenitor level. We have previously demonstrated that epigenetic age predictions in patients upon allogeneic hematopoietic stem cell transplantation correlate with donor age, while the microenvironment of elderly patients did not impose significant effects on age predictions [17]. This is in line with findings of this study, indicating that the epigenetic clock is hardly affected by a faster aging xenogeneic environment. It is yet unclear how lineage-specific or age-associated DNAm changes are controlled. Additional studies should be performed with longer time intervals after transplantation or with older mice (e.g., 50–100-week-old mice) to better understand how the aging environment impacts on epigenetic age. However, at much later time points after transplantation, the majority of human cells might shift toward expanded T cells, which would also be of interest with regard to lineage-specific DNAm changes in humanized mice. Our study opens the perspective to investigate how the cellular microenvironment can be modified to facilitate better lineage-specific epigenetic maturation—to ultimately understand how hematopoietic cell fate decisions are regulated.

## Materials and methods

### Humanized mice

Human hematopoietic stem and progenitor cells were isolated from human CB provided by the DKMS Cord Blood Bank Dresden. Three individual cord blood samples were pooled before Ficoll-Hypaque density centrifugation and magnetic enrichment for CD34^+^ cells according to the manufacturer’s instructions (Miltenyi Biotech) [6]. Fifty thousand CD34^+^ cells were injected intravenously in 150 μl PBS/5% FCS into five 7- to 9-week-old unconditioned KIT-deficient NSGW41 mice. After transplantation, mice were given neomycin-containing drinking water (1.17 g/l) for 3 weeks as described before [18].

### Flow cytometry and cell sorting

Nineteen weeks after transplantation, BM was collected from HuMice and prepared as described before [6]. Leukocyte counts of murine (mCD45^+^, clone 30F11; eBioscience) or human (hCD45^+^, clone HI30; BioLegend) origin were determined. Furthermore, cells were stained using anti-human antibodies for CD3 (clone OKT3), CD19 (clone HIB19), CD33 (clone WM-53), CD235 (clone HIR2; all eBioscience), CD16 (clone 3G8; BioLegend), and CD14 (clone M5E2; BD Biosciences) and the anti-mouse antibody Ter119 (clone TER-119; eBioscience). Blocking reagent used was ChromPure mouse IgG (Jackson ImmunoResearch). Flow cytometric measurements were performed on a LSRII cytometer (BD Biosciences) and analyzed using FlowJo software (TreeStar). Sorting of human CD45^+^ cells was performed on a FACSAriaTM II (BD Biosciences).

### Analysis of DNA methylation profiles

Genomic DNA was isolated from 10^6^ sorted human CD45^+^ cells using the QIAmp DNA Blood Mini Kit (Qiagen) according to the manufacturer’s instructions including RNA digest. DNA isolated from BM of humanized mice was bisulfite converted with the EZ DNA Methylation Kit (Zymo Research) according to the manufacturer’s instructions. DNAm profiles were subsequently analyzed with the Infinium HumanMethylation450 BeadChip (Illumina). This platform features more than 450,000 cytosine guanine dinucleotides (CpG sites). DNAm levels at individual CpG sites are provided as *β* values ranging from 0 (no methylation) to 1 (100% methylation). Raw data are accessible at Gene Expression Omnibus, www.ncbi.nlm.nih.gov/geo/, under the accession number GSE103010.

### Bioinformatics

Our DNAm profiles of HuMice were compared with our previous data on human CD34^+^ CB cells (*n* = 3; GSE40799) [8], B cells (*n* = 6; GSE35069) [7], and various human hematopoietic subsets that were isolated from peripheral blood (granulocytes, neutrophils, eosinophils, CD4^+^ T cells, CD8^+^ T cells, NK cells, and monocytes; *n* = 6 per cell type; GSE35069) [7]. For comparative analysis of epigenetic age predictions, we used DNAm profiles of human CB cells (*n* = 20; GSE30870, and *n* = 3; GSE40799) [8, 19], peripheral blood of 5-year-old individuals (*n* = 15; GSE83334) [20], 21–32-year-old individuals (*n* = 16; GSE65638) [21], and 89–103-year-old individuals (*n* = 20; GSE30870) [19]. All of these DNAm profiles were generated on the same Infinium HumanMethylation450 BeadChip platform.

For further analysis, CpG sites located on the X and Y chromosomes were excluded, missing values were estimated by k-nearest-neighbor (kNN) imputation, and data was quantile normalized. Unsupervised hierarchical clustering according to Euclidean distance and principal component analysis (PCA) were calculated in R. To estimate significant differences in DNAm, we applied limma paired *t* test in R (adjusted for multiple testing). *P* < 0.05 was considered as statistically significant. In addition, we selected for CpGs with a difference in mean *β* values > 0.2 or <− 0.2 in HuMice versus CD34^+^ CB samples to focus on CpGs with the highest difference. Functional classification of corresponding genes was performed with the GoMiner tool [22]. Enrichment of specific categories was calculated by the one-sided Fisher’s exact *P* value (not corrected for multiple testing) using all genes represented on the array as a reference. Epigenetic blood cell counts were calculated with the *Minfi* package in R using the *estimateCellCounts* function [9, 23]. For selection of B cell-specific CpGs, we filtered for CpGs with a difference in mean *β* value of > 0.7 or < − 0.7 in B cells (GSE35069) compared to other mature hematopoietic cell types isolated from peripheral blood (granulocytes, neutrophils, eosinophils, CD4^+^ T cells, CD8^+^ T cells, NK cells, and monocytes; GSE35069). Furthermore, only CpG sites with a standard deviation (SD) < 0.1 within the respective cell type(s) were considered as described before [10]. Using this selection strategy, 330 B cell-specific CpG sites were identified. To estimate donor age based on DNAm, three different epigenetic age predictors were applied as described by Weidner et al. [14], Hannum et al. [15], and Horvath [16]. Statistical significance of deviations of predicted and chronological age was estimated with the two-tailed, unpaired Student’s *t* test.

## Abbreviations

BM: Bone marrow
CB: Cord blood
DNAm: DNA methylation
HuMice: Humanized mice
